# Comparative Performance of the CamPROBE Local Anaesthetic Transperineal Biopsy Device Versus an In-Line Device for Detection of Significant Prostate Cancer

**DOI:** 10.3390/jcm14165702

**Published:** 2025-08-12

**Authors:** Kieran Sandhu, Syed Shah, Hannah Thorman, Kasra Saeb-Parsy, Vincent J. Gnanapragasam, Saiful Miah, Adham Ahmed

**Affiliations:** 1 Department of Surgery, University of Cambridge, Cambridge CB2 0QQ, UK; vjg29@cam.ac.uk; 2 Cambridge Prostate Cancer and Clinical Trials Group, Cambridge CB20QQ, UK; 3 Department of Urology, Cambridge University Hospitals, Cambridge CB2 0QQ, UK; s.shah2@nhs.net (S.S.); kasra.saeb-parsy@nhs.net (K.S.-P.); saiful.miah@nhs.net (S.M.); 4 Department of Urology, West Suffolk Hospital, Bury St Edmunds IP33 2QZ, UK; hannah.thorman@wsh.nhs.uk (H.T.); adham.ahmed@nhs.net (A.A.)

**Keywords:** prostate cancer, prostate biopsy, local anaesthetic transperineal biopsy

## Abstract

**Background/Objective:** The CamPROBE device offers a simple, low-cost method to facilitate double-free-hand local anaesthetic transperineal prostate biopsies (LATPBx). Here we present data from prospective implementation of its use for first biopsy cancer detection. **Methods**: The outcomes of two centres who adopted the CamPROBE were compared to a retrospective series of biopsies using an in-line (single-free-hand) device. All biopsies were done by clinicians new to the device. Outcomes were the detection of any cancer and clinically significant prostate cancer (csPCa) defined as ≥Grade Group 2 and ≥Grade Group 3 (GG2/3) and composite ≥NICE Cambridge Prognostic Group 2 and 3 (CPG2/3), as well as sampling accuracy of MRI-defined lesions. **Results:** Device cohorts were well matched for pre-biopsy PSA, T stage, and MRI visibility in both centres. In centre 1 (100 CamPROBE vs. 97 in-line biopsies), there were no differences in detection of any cancer or csPCa: ≥GG2 60.0% vs. 56.7% (*p* = 0.64), ≥GG3 31% vs. 20.6% (*p* = 0.09), ≥CPG2 62.0% vs. 60.8% (*p* = 0.86), and ≥CPG3 (*p* = 0.55). There were also no differences between devices in target biopsy positivity: 67.4% vs. 63.5% (*p* = 0.59). Data from centre 2 (38 CamPROBE vs. 44 in-line) re-capitulated these findings. The MRI target detection rate of the CamPROBE (assessed in Centre 1) was not affected by prostate volume, lesion laterality, anatomical position, or lesion size. Limitations include modest sample sizes, lack of randomization, and patient-reported outcomes. **Conclusions**: These data demonstrate that the CamPROBE device is a highly effective method of performing prostate biopsies with excellent cancer detection rates and accuracy, supporting its wider dissemination and use.

## 1. Introduction

Transperineal (TP) biopsy has emerged as a safe and effective means of achieving a histological diagnosis of prostate cancer (PCa) under local anaesthesia (LA) [1,2,3,4]. The use of local anaesthetic transperineal prostate biopsies (LATPBx) in the outpatient setting is expanding significantly as there is an international movement to replace transrectal biopsies with the TP approach [1,2,3,4,5,6].

The most prevalent current method for LATPBx uses the single-free-hand (SFH) technique facilitated by devices that are clipped on or attached to the ultrasound (US) probe (e.g., PrecisionPoint™, SureFire^®^). Biopsies are therefore performed “in-line” with the probe position. Beforehand, a separate deep infiltration with LA is usually needed as the pelvic floor is punctured repeatedly [7]. Current in-line devices in use for TP biopsies are costlier than those previously used for transrectal biopsies [8].

The CAMbridge PROstate Biopsy DevicE (CamPROBE) is a patented, low-cost, versatile alternative to in-line LATPbx devices [7]. CamPROBE is based on the coaxial concept but specifically for the perineal anatomy and to facilitate prostate biopsies. It includes an integrated needle for LA delivery (no sperate infiltration) and is long enough to be deployed beyond the pelvic floor to minimise internal pelvic floor trauma and pain during biopsy acquisition. The CamPROBE facilitates the “double-free-hand” (DFH) biopsy approach, which allows access to the whole prostate without being limited by attachment to the US probe and hence also avoids gland deformation. The DFH method was in fact the original first description of the TP biopsy technique [5]. The CamPROBE device was specifically designed to refine, standardise, and improve the accuracy of the versatile DFH technique [9]. The CamPROBE can be angled to reach different areas of the prostate and guide the biopsy needle to a target without biopsy needle deflection or repeated re-puncture of the pelvic floor.

While many TP biopsy devices have been described in the literature, to date, there have been very few comparisons between them in terms of cancer detection rates or biopsy accuracy. This is particularly salient as it has been opined by others that the CamPROBE-supported DFH method may be less accurate than in-line techniques [10,11]. Here, we compared key cancer detection and performance metrics in two centres that transitioned from an in-line device to the CamPROBE.

## 2. Patients and Methods

### 2.1. Cohort Assembly

**Figure 1:**
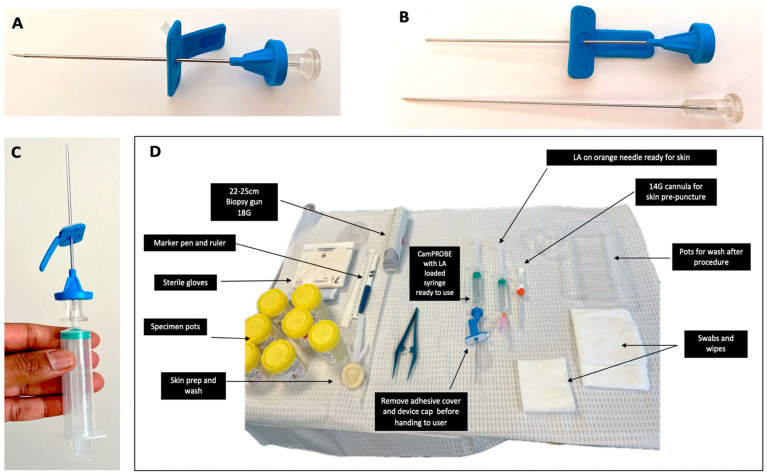
(**A**) The CAMbridge PROstate Biopsy DevicE (CamPROBE) device. (**B**) CamPROBE disassembled to show the integrated needle for local anaesthetic infiltration. Note the blunt-ended cannula within which the biopsy needle will sit; once deployed, this minimises internal cutting and shearing during device movement to acquire biopsies. (**C**) Illustration of syringe attached and ready for use. (**D**) Typical setup of the equipment and table for prostate biopsies. A video demonstration of the biopsy procedure is available at https://www.youtube.com/watch?v=tA-8DWOMjKM (accessed on 1 June 2025).

This was a prospective study of the diagnostic outcomes of 2 centres where 3 new users adopted the CamPROBE (JEB Technologies Ltd., Mildenhall, Suffolk, UK) (Figure 1) for prostate biopsies compared to a retrospective series of LATPBx performed using an in-line attached device (PrecisionPoint™ [PP] Perineologic, Cumberland, MD, USA) (2024–2025). Our specific focus comprised first-biopsies in men with suspected prostate cancer with no other exclusions for patient selection. There were no cases where the CamPROBE was not suitable for biopsy acquisition. Data from centre 1 formed the primary cohort, and data from centre 2 formed the validation cohort. This project had IRB approval from each centre (ID6620 PRN12620 and 5750, respectively).

### 2.2. Biopsy Procedure

CamPROBE procedures were carried out by three urologists new to the device but with extensive prior experience with the in-line device technique (PP): SM and SS in the primary cohort and AA in the validation cohort. For CamPROBE, all 3 attended dry-lab training (3h) followed by on-site mentoring for 3–4 cases, followed by independent practice. Cases assembled for this series included only those performed independently by SM, SS or AA. All patients had a pre-biopsy MRI and a combination of cognitive guided image targeting (based on MRI) and systematic biopsies using a sectoral sampling method as previously reported [12]. If there were no target lesions, then only systematic biopsies were performed. For both CamPROBE and the in-line device, the same sectoral sampling method was used (2 samples each for left and right anterior, middle, and base), with 2–4 taken from MRI defined target areas (if present). The in-line biopsy method is well reported and has been used at our centre since 2020 [10]. The CamPROBE biopsy technique is as described before, with some technical modification, and a video demonstrating its use is available at https://www.youtube.com/watch?v=tA-8DWOMjKM (accessed on 1 June 2025) [7]. Figure 1 shows the device details and the typical table setup for a biopsy. With the patient in lithotomy, two points are marked 1.5 cm above the anal verge and 1.0–1.5 cm on either side of the midline. At each marked site, 1.0–3.0 mL of 1% lignocaine is injected into the skin. The integrated CamPROBE device is assembled as per manufactures recommendation and attached to a 10 mL syringe filled with 1% LA (Figure 1). A linear array ultrasonic transducer is placed in the rectum, and the prostate and perineal tissue are visualised. The BK E14CL4B linear transducer ultrasound probe and BK 3000 ultrasound machine were used in this study (GE healthcare^®^, Burlington, MA, USA). Starting on the patient’s left, a skin-only pre-puncture is made in the pre-anesthetised area using a 14G cannula, and the CamPROBE is inserted into the skin breach under vision. The device is then advanced through the perineal fat until it reaches the pelvic floor. LA is delivered to superficial and deep perineal muscle in targeted small boluses (2–3 mL) using the integrated delivery needle as the device is passed through the pelvic floor to reach the prostate capsule. Once in position, the integrated needle is removed, leaving the coaxial access cannula in place. Through this cannula, standard 18G biopsies were taken following the pre-determined biopsy template using the DFH fan technique. As the prostate is insensate, further LA is usually not required at this step. The ultrasonic probe is rotated and angled to visualise the echogenic cannula and needle direction for biopsy acquisition from different sites. This was repeated on the contralateral side. In the primary cohort, total LA use was a median of 16 mls (range 8–26 mls).

### 2.3. Variables Collected and Data Analysis

Hospital electronic medical records (EMRs) were reviewed from each centre, and data were extracted for the variables of interest. Data was collected for baseline age and pre-biopsy prostate volume (PV), PSA at diagnosis, and MRI details (Table 1 and Table 2). Our primary aim was to assess cancer detection rates; hence, outcomes of any cancer and clinically significant cancers (csPCa) were recorded. Different endpoints for significant cancer (≥Grade Group [GG] 2, ≥GG3, and composite prognosis group ≥ Cambridge Prognostic Group [CPG] 2 and ≥ CPG3) based on the UK NICE guidelines were used to compare between devices [13]. The CPGs represent a composite prognostic stratification system that has been shown to more accurately predict prognosis for a diagnosis of prostate cancer [14]. In centre 1, we also collated data on more granular details for target biopsy accuracy including MRI-defined lesion laterality, size, location, and biopsy core length (Table 3). If there were 2 lesions in a prostate, we used data from the dominant/largest lesion. Differences in continuous measurements were assessed using a paired *t*-test for comparison of means, and the chi-squared test was employed for all other comparisons using proprietary software.

## 3. Results

### 3.1. Primary Cohort Characteristics and Cancer Diagnosis Rates

Data from 197 first biopsy procedures in the primary cohort were analysed from 100 men who underwent CamPROBE and 97 who had biopsies with the in-line device (Table 1). Between the CamPROBE and in-line cohorts, median PSA (*p* = 0.72), distribution of T1–T2 vs. T3–T4 (*p* = 0.92), and proportions with MRI visible lesions (*p* = 0.78) were well-matched. Most cases in both cohorts also had MRI Likert 4–5 lesions (Table 1). Targeted biopsy positive rates were similar between the two cohorts at 67.4% vs. 63.5% (*p* = 0.59), with no differences when MRI lesions were stratified by Likert score (Table 1). Cancer detection for any grade was similar for both the CamPROBE and in-line devices (78% vs. 79%, respectively; *p* = 0.81) (Table 1). Using different definitions of clinical significance, we also found no differences in detection of csPCa. For ≥GG 2, detection rates were 60% and 56.7%, respectively (*p* = 0.64) and detection of ≥GG 3 was 31% and 20.6% (*p* = 0.09). Given that the CPG classification is used in deciding treatment options, we also looked at detection based on this criterion [13]. We again found no differences in prognostic group allocation between the two cohorts: ≥CPG2 was 62% vs. 60.8% (*p* = 0.86) for CamPROBE vs. in-line devices and 37% vs. 32.9% (*p* = 0.55) for ≥CPG3, respectively (Table 1).

### 3.2. Validation Cohort Characteristics and Cancer Diagnosis Rates

Data from 82 first biopsy procedures in the validation cohort were analysed: 38 had CamPROBE biopsies, and 44 men had the in-line device (Table 2). Both cohorts were again well matched for the following pre-biopsy clinical–pathological characteristics: median PSA (*p* = 0.95), T1–T2 vs. T3–T4 (*p* = 0.95), and proportions of MRI-visible lesions (*p* = 0.76) (Table 2). Targeted biopsy positivity rates were very similar (81.8% CamPROBE vs. 63.4% in-line) (*p* = 0.08). Any cancer detection was similar for both the CamPROBE and in-line devices (84.2% vs. 84%, respectively, *p* = 0.98) (Table 2). For detection of ≥GG2, detection rates were 73.6% and 68.1% (*p* = 0.58), and for ≥GG3, they were 50% vs. 27.2% (*p* = 0.03). Detection of ≥CPG2 was also similar at 76.3% vs. 75% (*p* = 0.96), and for ≥CPG3, it was 55.2% vs. 34% for CamPROBE vs. the in-line device group (*p* = 0.05).

### 3.3. Device Accuracy Analysis

A critical attribute of any LATP device is to facilitate accurate spatial placement of biopsies for diagnosis. To assess this, we looked for sampling accuracy in MRI-defined targets in our primary cohort as a measure of device performance. A total of 83 men each in the CamPROBE and in-line device group had information on MRI lesions that were targeted for biopsies (Table 3). We had previously noted no overall differences in target biopsy positivity rates between device cohorts (Table 1). We also found that laterality of MRI lesion and lesion location in the prostate did not affect the performance of either device (Table 3). Prostate volume (PV) also did not affect the performance of the CamPROBE device with no differences in target positivity for glands ≤ 50 or >50 mls (69.5% vs. 62.5%, *p* = 0.53). The in-line device however appeared to be less accurate, with larger PVs (79.6% vs. 38.2%, *p* = 0.0003) (Table 3). Comparison between device groups showed no difference for PVs ≤50 mls (*p* = 0.23), but for PVs > 50 mls, it trended to be better with CamPROBE (*p* = 0.06). When the cohorts were stratified by MRI-reported lesion size, we observed that with the in-line device, target positivity rates were higher with lesions > 100 mm^2^ compared to smaller lesions (72.7% vs. 48.8% *p* = 0.017). In contrast, target positive rates were not affected by lesion size when using the CamPROBE device (*p* = 0.14) (Table 3). Finally, we also compared the quality (measured by core length) of the biopsy core extracted by each device group and found no differences in core lengths (median of 7 mm with CamPROBE vs. 6.5 mm for the in-line device, *p* = 0.68).

### 3.4. Comparison with Other Biopsy Studies

Many studies have reported on LATPBx cancer detection outcomes using in-line devices. To benchmark our results from CamPROBE, we compared any cancer and ≥GG 2 detection rates between reported studies on MRI-guided first biopsy procedures (Table 4). CamPROBE demonstrated equivalent overall and ≥GG 2 cancer detection rates compared to a single-centre study using PP (70.4% and 49.6%, respectively) [10]. Similarly, cancer detection rates were comparable to 2 large multi-centre studies using either PP or a combination of different in-line devices (Table 4) [3,15]. Finally, we also compared our results with a multi-centre study by Hansen et al. who used GA template grid-based biopsies (considered the gold standard) [16]. Here, again, we found very comparable detection rates with our CamPROBE primary and validation cohort results for detection of any cancer and ≥GG 2 cancer (Table 4).

## 4. Discussion

This study demonstrates that the CamPROBE device-supported DFH method is a highly effective method of performing LATPBx and yields cancer detection rates similar to in-line methods. We further found that the CamPROBE retained accuracy regardless of prostate size, MRI lesion location, or MRI lesion size. In this study, all cases were performed by clinicians new to the device who had attended standard biopsy training and mentoring without the need for a long learning curve. The ease of adoption of the DFH method is further proven by the findings of Honore et al., who transitioned from TRUSBx to LATPBx in their centre using a simplified CamPROBE method with a short learning curve, minimal complications, and excellent cancer detection rates [1]. Thus, the opinions expressed in the previous literature that the CamPROBE-enabled DFH method is hard to learn, difficult to co-ordinate with US, and may result in lower cancer detection rates is unfounded [11,15].

As the variety of LATPBx devices increase there is a need to benchmark devices against one other. To our knowledge, there has been no previous direct comparison of different devices. One recent study did look at the DFH vs. SFH approach in a single-institution training programme [7]. The DFH method described however was to directly insert the flexible unsupported biopsy needle into the perineum without a device or coaxial cannula to support it. Compared to an in-line device, they reported poorer csPCa (≥GG2) detection rates and biopsy core lengths. Our data has in contrast found no difference in either ≥GG2 cancer detection rates or biopsy quality using the DFH approach enabled by the CamPROBE device. Indeed, we observed that the CamPROBE DFH method may potentially be better than in-line devices when the prostate is large or MRI lesions smaller. The excellent cancer detection and accuracy of the coaxial-supported DFH method have also been reported by others in much larger datasets [1]. Thus, we would not recommend the use of the DFH method unless it is used in conjunction with a device specially designed to facilitate this method (just like the SFH method requires the use of specific devices).

We believe the CamPROBE offers many advantages over other prostate biopsy devices. It is easily adoptable, requiring minimal training and a short learning curve for clinicians transitioning from in-line devices or the transrectal prostate biopsy method. The DFH method used with the CamPROBE device provides greater freedom to access zones typically difficult to reach, especially the anterior/apical regions of the prostate. It facilitates this without deformation from the probe attachment, which may improve sampling accuracy and likely increase patient comfort. The CamPROBE coaxial cannula helps to stabilise the biopsy needle path, which reduces deflection, aiding in the targeting of small or anterior lesions. In our study, we utilised the BK US machine (General Electric Healthcare^®^, Chicago, IL, USA) but unlike other in-line systems that require bespoke probe adaptors or taping to prevent the device sliding around, CamPROBE’s independence from any specific US device or vendor simplifies logistics, streamlines setup, and enhances scalability across institutions with varying infrastructure. The CamPROBE’s echogenic long cannula length circumvents deep structure/pelvic floor re-puncture when the biopsy needle is inserted to acquire biopsies. This is often a point of significant pain for patients, and in-line devices necessitate pre-puncture infiltration of between 20 and 30 mls of LA into the pelvic floor [17,18]. The CamPROBE coaxial design and integrated LA delivery system further allow for more anatomical placement and hence reduced use of LA (median 16 mls in the primary cohort), which is ultimately safer for patients. The CamPROBE offers further significant benefits in terms of its cost, with a UK price of £47 in contrast with in-line devices, which range from £120 to £220 [19]. This is likely to mean that the CamPROBE offers excellent cost–benefit ratios and savings in an ever more cash-strapped NHS and global health system [20]. The fast setup time, low local anaesthetic volume, and reduced disposable costs may also offer long-term sustainability benefits for an increasingly pressured global healthcare system.

Whilst this study has demonstrated equivalence in cancer detection rates between CamPROBE and other in-line biopsy devices, there are natural limitations to our study, with a modest sample size in each centre and non-randomisation between devices. Our modest sample size limits the power of this study to detect small changes, and findings such as the better ≥GG3 detection trend require further verification in larger cohorts for accurate interpretation. The primary goal here however was to report on our initial experience and findings as the first ever study to compare performance of the CamPROBE device with any other transperineal device. The findings of this study (detection rates and ranges) will now be used to inform power calculations for a future prospective trial to see if any differences truly exist between performances of different devices. We have here only compared performance between CamPROBE and one in-line SFH device, but we do not have any reason to suspect that any other in-line device would be different as they all share the same method and technique (e.g., Surefire, Koelis, Leapmed). Ultimately, to show superiority (or non-inferiority) of one device over another will require a well powered randomised trial. We also did not collect data on side effects, complications, or patient-reported outcomes. However, we have previously reported this for CamPROBE in our single- and multi-centre study, where we found no infections, device deficiencies, or safety issues and excellent patient tolerability and acceptability [7,21]. We further recognise another limitation in the fact that this study used retrospective historical controls as we were charting our experience of moving from one device to another. While not ideal, the use of historical controls can serve an audit benchmark for introduction and calibration when a new device or technique is introduced into clinical practice. Therefore, when used, it is important to ensure the historical retrospective and prospective cohorts were as comparable as possible. In this study, both cohorts were well-matched for key clinico-pathological variables such as PSA, MRI stage, and Likert scores. In addition, the same sampling template and operators were used across both groups. While these facts do help mitigate against potential selection bias, we acknowledge that there may always be other unknown factors that could result in error. These can only be addressed with matched larger prospective series and especially randomised studies in this area, as detailed above, and these are in the planning.

## 5. Conclusions

CamPROBE is an effective, reliable means of performing LATPBx and shows cancer detection rates equivalent to those of current in-line devices. Our data supports the use of the CamPROBE as a safe, simple, low-cost, and accurate device for clinicians to use to perform LATPBx for suspected prostate cancer.

## Figures and Tables

**Table 1:** Baseline characteristics and cancer detection outcomes of the primary cohort between patients undergoing CamPROBE and in-line device prostate biopsies.

	CamPROBE n = 100	In-Line Device n = 97	
**Age (y)**			*p* = 0.01
Mean	66	69	
Median	65	69	
Interquartile range	61–73	63–75	
**PSA (ng/mL)**			*p* = 0.72
Mean	25.8	21.5	
Median	6.1	7.4	
Interquartile range	4.1–12.4	4.9–11.6	
**Prostate volume (mls)**			*p* = 0.02
Mean	44.9	52.7	
Median	39.5	46.7	
Interquartile range	30–53.8	33–63	
No data	6	0	
**MRI stage (pre–biopsy)**			*p* = 0.92
T1–T2	78	81	
T3–T4	14	14	
Metastatic	n = 8	n = 2	
**MRI Likert score**			*p* = 0.78
1–2	10	9	
3	8	7	
4–5	75	78	
No data available	n = 7	n = 3	
**Target positivity (%)**			*p* = 0.59
**All MRI lesions**	56/83 (67.4%)	54/85 (63.5%)	
Likert 3	2/8 (25.0%)	2/7 (28.6%)	
Likert 4	28/45 (62.2%)	22/42 (52.3%)	
Likert 5	26/30 (86.7%)	30/36 (83.3%)	
**Cancer detection (%)**			
Any cancer	78%	79%	*p* = 0.81
≥Grade Group 2	60%	56.7%	*p* = 0.64
≥Grade Group 3	31%	20.6%	*p* = 0.09
≥Cambridge Prognostic Group 2	62%	60.8%	*p* = 0.86
≥Cambridge Prognostic Group 3	37%	32.9%	*p* = 0.55

**Table 2:** Baseline characteristics and cancer detection rates of the validation cohort between patients undergoing CamPROBE and in-line device prostate biopsies.

	CamPROBE n = 38	In-Line Device n = 44	
**Age (y)**			*p* = 0.15
Mean	68.1	70.6	
Median	67.5	71.5	
Interquartile range	63–76	66–76	
**PSA (ng/mL)**			*p* = 0.95
Mean	11.3	11.1	
Median	7.9	8.3	
Interquartile range	5.9–14.9	7–12.5	
**Prostate volume (mls)**			*p* = 0.14
Mean	44.3	51.3	
Median	41.5	47	
Interquartile range	32.7–58.7	34.9–67.7	
**MRI stage (pre-biopsy)**			*p* = 0.95
T1–T2	28	34	
T3–T4	8	10	
**MRI Likert score**			*p* = 0.76
1–2	2	3	
3	6	8	
4–5	30	33	
**Target positivity (%)**			*p* = 0.08
**All MRI lesions**	27/33 (81.8%)	26/41 (63.4%)	
Likert 3	2/4 (50.0%)	2/8 (25.0%)	
Likert 4	9/11 (81.8%)	7/12 (58.3%)	
Likert 5	16/18 (88.8%)	17/21 (80.9%)	
**Cancer detection (%)**			
Any cancer	84.2%	84.0%	*p* = 0.98
≥Grade Group 2	73.6%	68.1%	*p* = 0.58
≥Grade Group 3	50%	27.2%	*p* = 0.03
≥Cambridge Prognostic Group 2	76.3%	75.0%	*p* = 0.96
≥Cambridge Prognostic Group 3	55.2%	34.0%	*p* = 0.05

**Table 3:** Analysis of target detection rates of biopsies taken by either CamPROBE or the in-line device in the primary cohort.

Number with MRI targets	CamPROBE	In line device	
**Target positivity by laterality** RIGHT LEFT BOTH/DIFFUSE	**n = 83** 27/42 (64.3%) 18/24 (75.0%) 11/17(64.7%) Within group comparison *p* = 0.36	n = 83* 26/44 (59.0%) 19/31 (61.2%) 8/8 (100%) Within group comparison *p* = 0.84	*p* = 0.62 *p* = 0.28 *p* = 0.12
**Target positivity by lesion location ^#^** Anterior Posterior Lateral Diffuse/extensive	**n = 83** 7/11 (63.6%) 14/23 (60.9%) 29/43 (67.4%) 6/6 (100%) Within group comparison *p* = 0.86	n = 83 * 8/13 (61.5%) 20/33 (60.6%) 20/33 (60.6%) 4/4 (100%) Within group comparison *p* = 0.99	*p* = 0.91 *p* = 0.98 *p* = 0.54 NA
**Target positivity by** **prostate size (mls)** Prostate ≤50 with target Prostate >50 with target	**n = 83** 41/59 (69.5%) 15/24 (62.5%) Within group comparison *p* = 0.53	n = 83 * 39/49 (79.6%) 13/34 (38.2%) Within group comparison *p* = 0.0003	*p* = 0.23 *p* = 0.06
**Target positivity by** **MRI lesion size (mm^2^) ^#^** Lesion size ≤100 Lesion size >100	n = 76 ** 26/44 (59.0%) 24/32 (75.0%) Within group comparison *p* = 0.14	n = 76 ** 21/43 (48.8%) 24/33 (72.7%) Within group comparison *p* = 0.017	*p* = 0.33 *p* = 0.94
**Biopsy core length** **from positive target (mm) ^#^** Mean Median Interquartile range	**n = 56** 7.41 7 4–11	**n = 52** 7.0 6.5 3–10	*p* = 0.68

* missing data in 2 cases; ** missing data in 7 cases; ^#^ where there were ≥2 lesions, the dominant/target lesion was included.

**Table 4:** Comparison of cancer detection rates between the CamPROBE biopsies in this study and other contemporary series using in-line devices.

Device for First Biopsy	Number	Overall Cancer Detection Rate (%)	≥Grade Group 2 Detection Rate (%)
**CamPROBE** **(this study)** Primary cohort Validation cohort	100 38	78 (78.0) 32 (84.2)	60 (60.0) 28 (73.7)
**In-line LATP device** Kum et al. 2020 [10] ^a^ Lopez et al. 2021 [15] ^a^ (multi-centre) Bryant et al. [3] ^b^ (multi-centre RCT-TP arm)	115 674 562	81 (70.4) 487 (72.0) 360 (69.4)	57 (49.6) 406 (60.2) 329 (58.5)
**GA grid-based TP biopsies** Hansen et al. [16] ^c^ (multi-centre)	807	546 (67.6)	392 (49.3)

^a^ PrecisionPoint™ device ; ^b^ PrecisionPoint™ or BK UA1232™ devices; ^c^ template grid-based biopsies under GA.

## Data Availability

Please contact the corresponding author for availability of the datasets used and/or analysed during the current study.
